# Tortuosity of the descending thoracic aorta: Normal values by age

**DOI:** 10.1371/journal.pone.0215549

**Published:** 2019-04-23

**Authors:** Viony M. Belvroy, Hector W.L. de Beaufort, Joost A. van Herwaarden, Jean Bismuth, Frans L. Moll, Santi Trimarchi

**Affiliations:** 1 Thoracic Aortic Research Center, IRCCS Policlinico San Donato, San Donato Milanese, Italy; 2 Department of Vascular Surgery, University Medical Center Utrecht, Utrecht, Netherlands; 3 Houston Methodist DeBakey Heart & Vascular Center, Houston, Texas, United States of America; 4 Department of Health and Community Sciences, University of Milan, Milan, Italy; 5 Fondazione IRCCS Ca’ Granda Ospedale Maggiore Policlinico Milan, Milan, Italy; Medical University Innsbruck, AUSTRIA

## Abstract

**Background:**

Aging changes the aorta in length, tortuosity and diameter. This is relevant in thoracic endovascular aortic repair (TEVAR) and in the long term follow up.

**Methods and results:**

Two groups of hundred patients < 65 years and hundred patients ≥ 65 years, with no vascular diseases were made. Thin cut CT scans were analyzed with 3Mensio Vascular software and the following measurements were collected; tortuosity index, curvature ratio, maximum tortuosity in degrees and the level of vertebrae of the maximum tortuosity. The descending thoracic aorta (DTA) was analyzed and was divided into four zones of equal length. Subjects were divided into three groups based on their maximum tortuosity value: low (< 30°), moderate (30° – 60°) and high (> 60°). A linear regression model was built to test the effect of age and gender on tortuosity. Tortuosity was more pronounced in the ≥ 65 compared to the < 65 group (tortuosity index: 1.05 vs. 1.14, respectively; p < 0.001), curvature ratio (1.00 vs. 1.01; p < 0.001), maximum tortuosity (22.24 vs. 27.26; p < 0.001), and group of angulation (low vs. low; p < 0.001). Additionally, the location of maximum tortuosity was further distal for the ≥ 65 group (level of vertebrae; 5.00 vs. 5.00; p < 0.001), and zone of maximum tortuosity (4A vs. 4A; p < 0.001). There was no significant difference between male and female subjects.

**Conclusion:**

Normal DTA tortuosity increases with age. This is important to understand natural aging and for TEVAR planning and follow-up.

## Introduction

As every stent graft has a fixed length and diameter, which allows only some oversizing, it is important to understand how the aorta changes in length, tortuosity and diameter during follow-up after thoracic endovascular aortic repair (TEVAR). This inevitable process of ageing may sabotage the initial excellent endovascular results. Hence, more knowledge about this physiological phenomenon might help to improve stent grafts designs in order to create durable results of endovascular repairs.

Aging of the healthy and diseased aorta is an irreversible process, of which a number of aspects have been clarified. First of all, the aorta may lengthen by itself[1–4]., or become longer as the vertebral column may decrease over time due to spinal shrinkage[5], inducing increased tortuosity of the descending thoracic aorta (DTA). Second, aging has been shown to be a substantial risk factor for the decrease of elastin and increase of collagen in the aortic wall[6]. Loss or malfunctioning of elastin structures is pernicious for the cardiovascular system[7,8]. As elastic tissue breaks down in the aortic wall, the amount of sclerotic tissue increases. Consequently, arteriosclerosis increases and the aorta becomes less compliant, which needs to be compensated with an increase of the systolic blood pressure, also called a decrease of the Windkessel effect[9]. In addition, a German study from 1977 shows that the aortic diameter increases with age[3,9]. According to Laplace’s law, wall tension is directly dependent on aortic diameter, so at an older age aortic wall tension is increased due to this phenomenon as well.

The Ishimaru classification gives a good view of the aortic arch and recently an additional classification has been published[10,11]. Zone 4, the descending thoracic aorta, is the longest zone, which can get angulated with aging. An update on this classification is therefore needed. Likewise, this stems out of the importance of TEVAR for the treatment of thoracic aortic diseases. The proximal and distal landing zones should be healthy and have ideally a diameter of <40 mm and length of >20 mm. It is recognized that higher angulation can result in an inadequate seal or migration of the stent graft, which can lead to endograft failure[12,13], and tortuosity has been identified as a risk factor for type 1b endoleak[14].

In order to define acceptable landing zones, the normal tortuosity of the descending aorta needs to be defined, which is the principal aim of this study. Therefore, we started to study the descending thoracic aorta of 200 non-vascular patients of different age, as age is likely to be an important confounding factor.

## Methods

### Patients

The local Ethical Committee of I.R.C.C.S. Policlinico San Donato approved of this retrospective study and waived the requisite to obtain informed consent from patients. Two hundred CT scans of adult patients who underwent diagnostic evaluation for various indications, like pulmonary diseases, at our institution in 2017 were selected at random. Patients with aortic aneurysm or dissection were excluded from this study, so only aortas without vascular diseases were selected. The patients were divided in two groups; 100 < 65y patients and 100 ≥ 65y patients. Patient demographics are summarized in Table 1.

**Table 1:** Baseline demographic characteristics of patients.

		< 65y (N = 100)	≥ 65y (N = 100)	p-value
Age (y)		50.94 +/- 6.68	78.40 +/- 7.80	< 0.001*
	Range	32–59	65–91	
Gender		N (%)	N (%)	
	Male	- 50 (50%)	- 50 (50%)	
	Female	- 50 (50%)	- 50 (50%)	
Type of arch		N (%)	N (%)	< 0.001*
	Type I	66 (66%)	38 (38%)	
	Type II	30 (30%)	43 (43%)	
	Type III	4 (4%)	19 (19%)	
Mean centreline length (cm)		190.7 +/- 16.5	212.8 +/- 22.2	< 0.001*

*P-value is significant when p < 0.05

### Image analysis & outcomes

Selection criteria for the image analyses were thin cut CT scan slices of 1.0 or 1.5 mm. Most scans did not have contrast, and were only selected when quality was good to perform the measurements. The type of arch was measured using the aortic arch classification[15].

**Fig 1:**
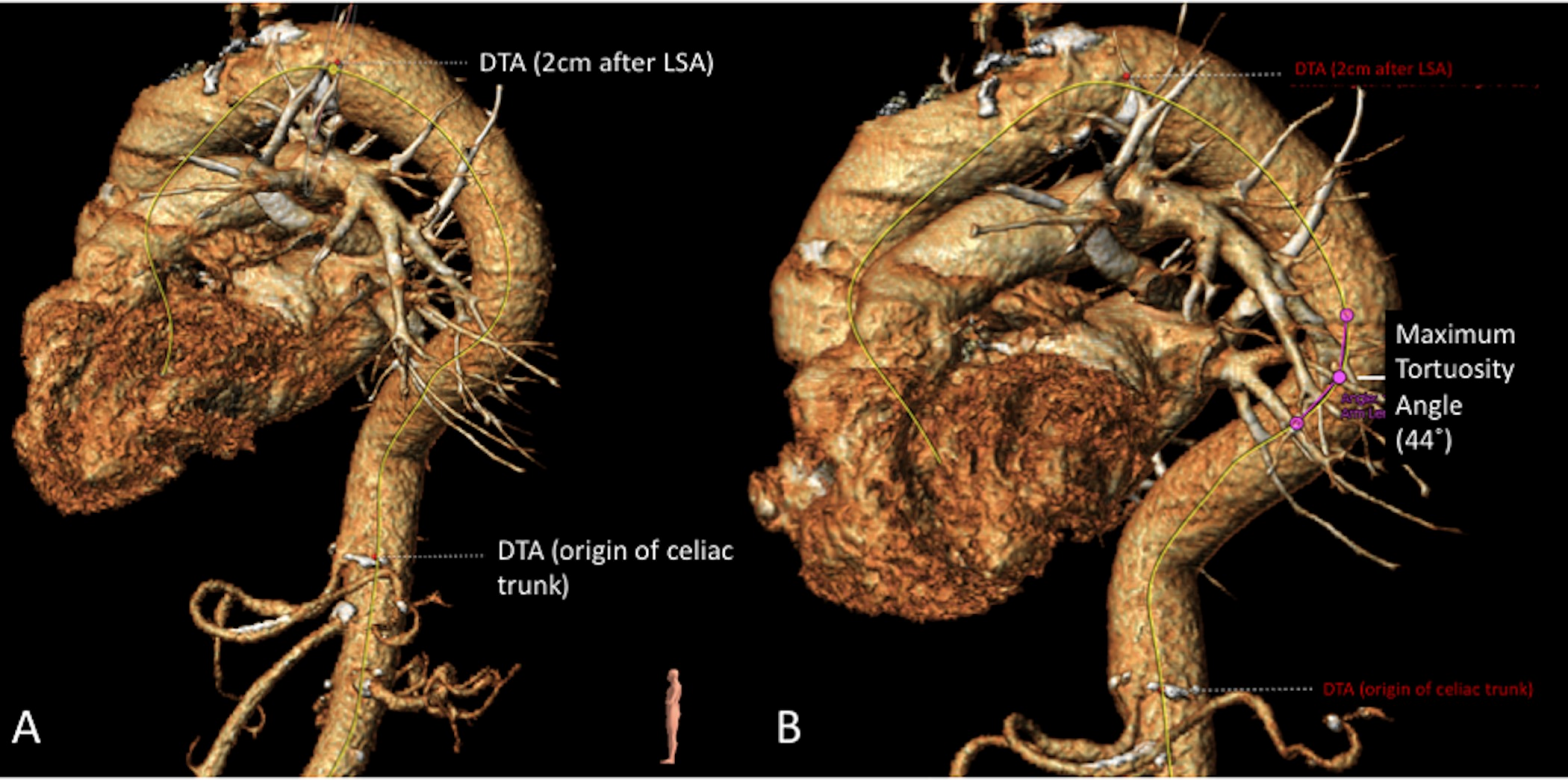
CT scan measurements performed in 3Mensio software. Two images of measurements in 3Mensio in the DTA; A: Center lumen line created for zone 4 (yellow line), with the start of the DTA 2 cm after the LSA up to the celiac trunk; B: *Maximum tortuosity angle* with two line elements that are defined by three control points over the centerline to measure the tortuosity in degrees.

The CT scans were analyzed with 3Mensio Vascular software (3Mensio Medical Imaging B.V., Bilthoven, The Netherlands). The measurements were performed in the DTA, where a center lumen line was created semi-automated from two centimeters after the left subclavian artery (LSA), where zone 4 starts in Ishimaru’s aortic map[10], up to the celiac trunk. The length of the center lumen line was automatically calculated. Two markers were placed at the beginning and at the end of the centerline. The functional *true length* was measured in a 3D model, which is the distance between the beginning and the end of zone 4 in a straight beeline. The *tortuosity index* was calculated by dividing the length of the center lumen line by the true length[16,17]. See Fig 1.

The tortuosity angle function of 3Mensio was used to measure the tortuosity in degrees over the centerline[18]. This angle is measured between two line elements that are defined by three control points over the centerline, see Fig 1, *the maximum tortuosity angle*. The first point is the start of the first line element, the second point is the end of the first and the start of the second line element. The third point is the end of the second line element. The distance between the first and the third was set at 15mm, a standard setting in 3Mensio. The maximum degrees of tortuosity was collected, together with the height of occurrence of this tortuosity. This was measured with the level of vertebras.

The *curvature ratio* was calculated by the outer curvature length of the descending thoracic aorta divided by the length of the center lumen line.

**Fig 2:**
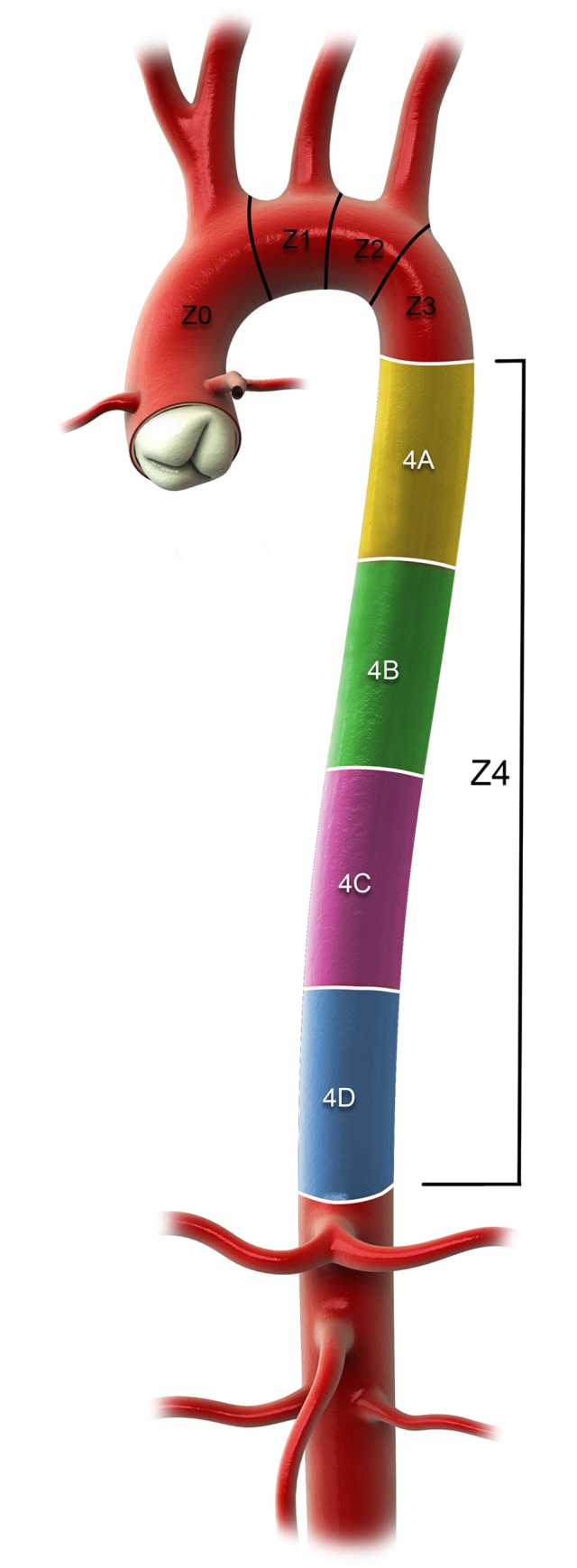
The four equal zones of the descending thoracic aorta– 4A to 4D.

**Fig 3:**
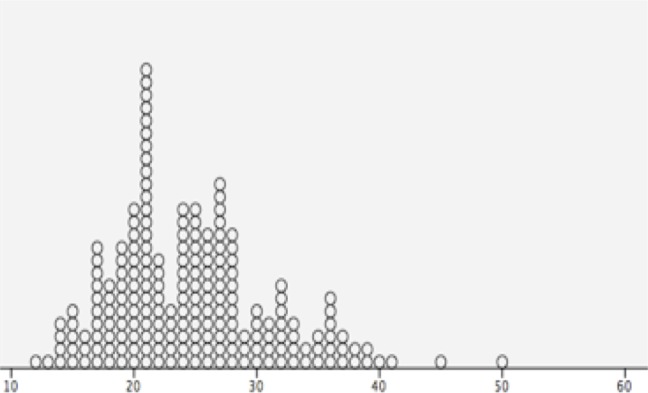
Scatterplot by SPSS software of the maximum tortuosity in degrees. This scatterplot shows how to divide the normal tortuosity in degrees. Most patients have a maximum tortuosity < 30°, so the cut-off point between low and moderate tortuosity is 30°.

**Fig 4:**
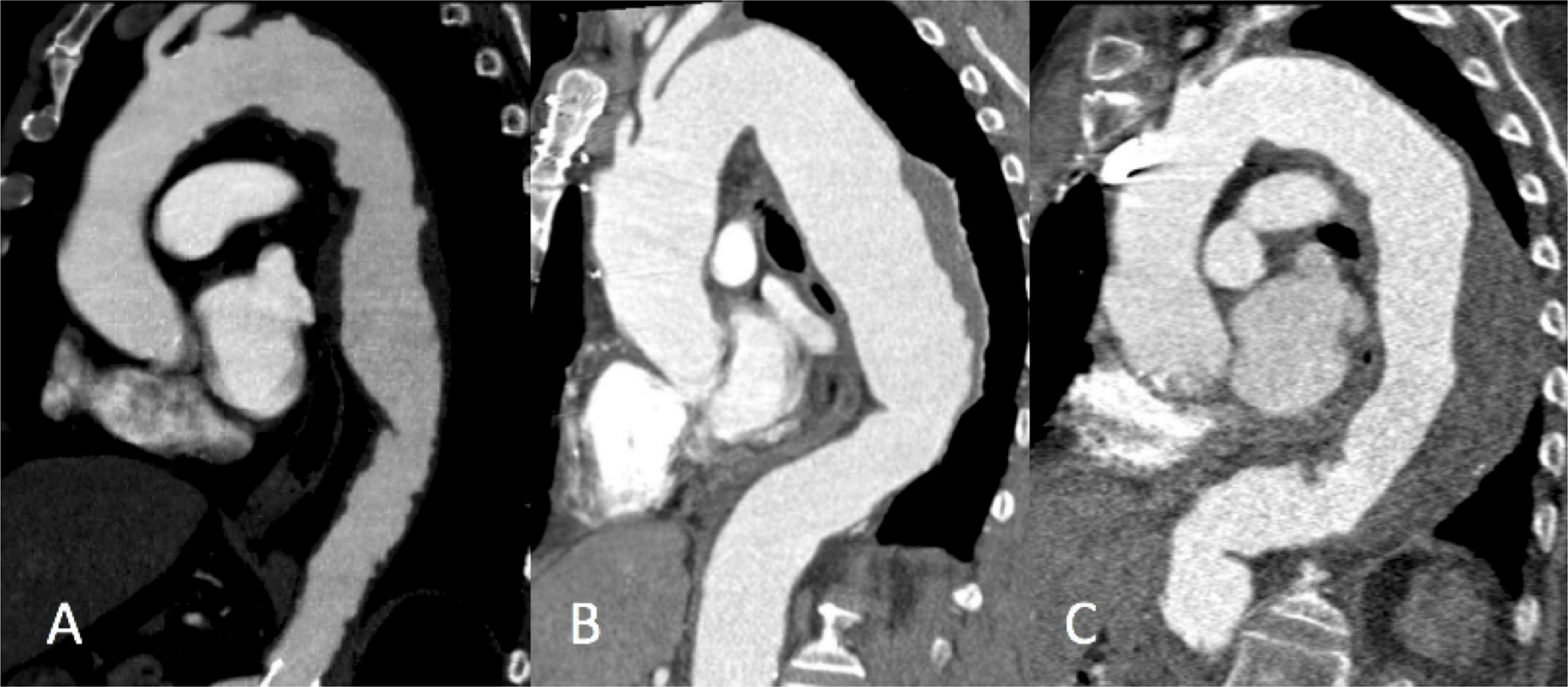
Three groups of amount of tortuosity. A: low tortuosity < 30°; B: moderate tortuosity 30° - 60°; C: high tortuosity > 60°.

The DTA was divided into four zones (see Fig 2). The centerline was divided in four equal parts and numbered from proximal to distal in *4A* to *4D*. The maximum tortuosity in degrees was put in a scatter plot to see how the tortuosity can be divided in groups (see Fig 3). The groups were divided as followed: low tortuosity < 30°, moderate tortuosity 30° – 60° and high tortuosity > 60°. See Fig 4.

Measurements were repeated for 40 scans (20%) randomly selected from the study group to assess intra-observer and inter-observer creditability (by V.M.B. & H.W.L.d.B.).

### Statistical analysis

The data was analyzed using SPSS Statistics 23 software (IBM Corp, Armonk, NY). Normality was tested with the Shapiro Wilk test. Comparison between the two groups was done with the independent sample T-test for normally distributed data or the Mann-Whitney U test for non-normally distributed data. The effect of age and gender on tortuosity was analyzed calculating a linear regression model. Intra- and inter observer variability was tested with the Intraclass Correlation Coefficient (ICC).

## Results

**Fig 5:**
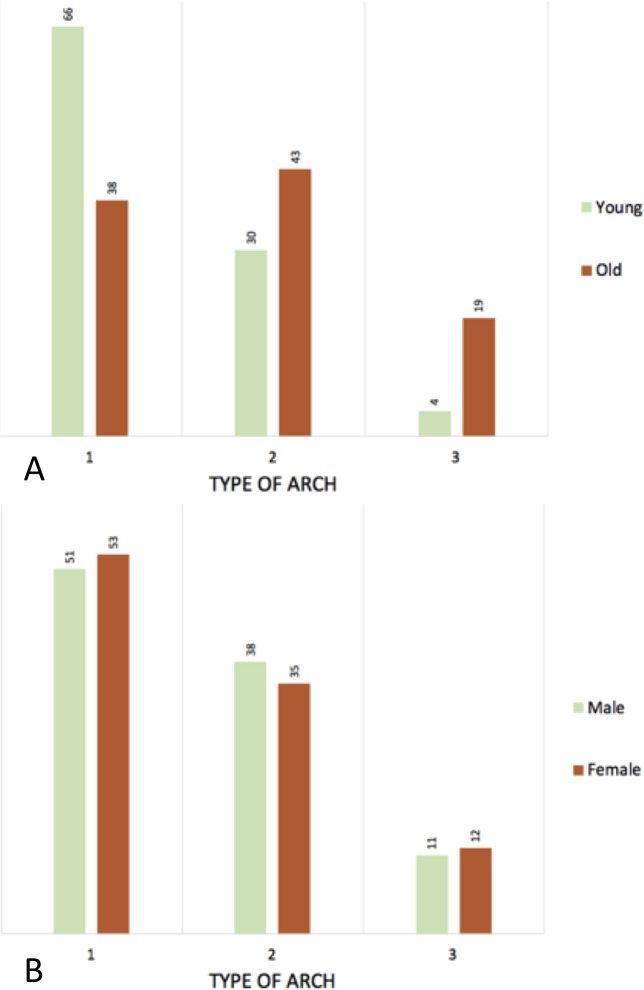
Histogram of the type of arch. A: young vs. old; B: male vs. female.

**Fig 6:**
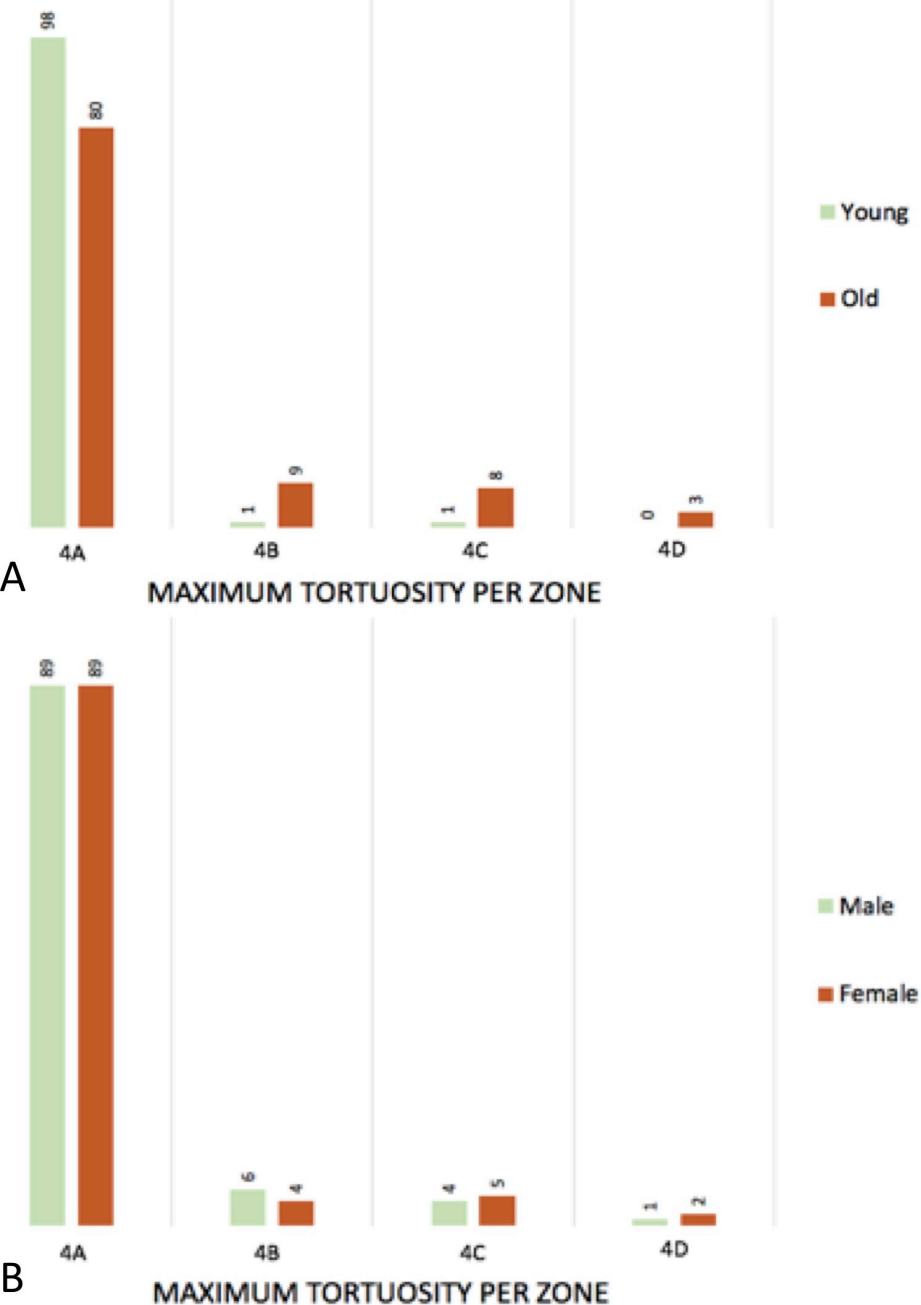
Histogram of the maximum tortuosity per zone. A: young vs. old; B: male vs. female.

The patients were split into two groups of 100 patients. The < 65y patients had a mean age of 50.9 years old and the ≥ 65y patients had a mean age of 77.9 years old. The type of arch was measured in all patients, type 1 (< 65y = 66; ≥ 65y = 38), type 2 (<65y = 30; ≥ 65y = 43) and type 3 (< 65y = 4; ≥ 65y = 19), see Fig 5. The mean length of the centerline differs between the groups (< 65y = 190.7cm vs. ≥ 65y = 212.5cm; 0.000), see Table 1. The differences were measured between the < 65y and ≥ 65y patients in tortuosity index (1.05 vs. 1.14; p = 0.000), curvature ratio (1.00 vs. 1.01; p = 0.000), maximum tortuosity in degrees (22.24 vs. 27.26; p = 0.000), the level of vertebrae of the maximum tortuosity (5.00 vs. 5.00; p = 0.001), the zone of maximum tortuosity (4A vs. 4A; p = 0,000), and the groups of angulation (low vs. low; p = 0,000). For an overview see Table 2 and Fig 6.

**Table 2:** Outcome measurements comparing patients young vs. old.

				< 65y (N = 100)	≥ 65y (N = 100)	p-value
				Mean (std.)	Mean (std.)	
Tortuosity index				1.05 (0.024)	1.14 (0.078)	< 0.001*
	CI^a^			1.05–1.06	1.13–1.16	
Curvature ratio				1.00 (0.105)	1.01 (0.012)	< 0.001*
	CI^a^			0.99–1.00	1.01–1.01	
Maximum Tortuosity				22.24 (4.710)	27.26 (7.233)	< 0.001*
	CI^a^			21.31–23.17	25.82–28.70	
Level of Vertebrae				5	5	0.001*
	Range			5–10	5–12	
Minimum Tortuosity				0.83 (0.753)	1.67 (1.386)	< 0.001*
	CI^a^			0.68–0.98	1.40–1.94	
Level of Vertebrae				11	11	0.032*
	Range			8–12	7–12	
Zone of maximum Tortuosity				4A	4A	< 0.001*
	Range			4A –D	4A – 4D	
Group of angulation				Low	Low	< 0.001*
	Range			Low–Moderate–High	Low–Moderate–High	
Percentage of patients with high tortuosity				0 (0%)	0 (0%)	

*P-value is significant when p < 0.05

^a^CI: Confidence Interval

After a stepwise linear regression analysis only one independent variable has a significant correlation with the tortuosity and the maximum angulation, which is age. With aging the tortuosity becomes higher, see also Table 3.

**Table 3:** Linear regression model of tortuosity index and maximum tortuosity in relation to age and gender.

			All patients (N = 200)		
Geometrical variable			β	P value	Model r
Tortuosity Index					
	Age		0.03	< 0.001	0.652
	Gender		0.06	0.424	0.654
Maximum Tortuosity					
	Age		0.175	<0.001*	0.405
	Gender		-0.393	0.647	0.406

*P-value is significant when p < 0.05

The same measurements were done comparing males with females. The type of arch in all patients was as followed; type 1 (Male = 51; female = 53), type 2 (male = 38; female = 35) and type 3 (male = 11; female = 12), see Fig 5. The measurements between males and females in tortuosity index (1.09 vs. 1.10; p = 0.626), curvature ratio (1.00 vs. 1.01; p = 0.411), maximum tortuosity in degrees (24.58 vs. 24.65; p = 0.960), the level of vertebrae of the maximum tortuosity (5.00 vs. 5.00; p = 1.000), the zone of maximum tortuosity (low vs. low; p = 0.821), and the groups of angulation (4A vs. 4A; p = 0.862). For an overview see Table 4 and Fig 6.

**Table 4:** Outcome measurements comparing patients male vs. female.

		Male (N = 100)	Female (N = 100)	p-value
		Mean (std.)	Mean (std.)	
Age (y)		63.88 +/- 14.381	64.98 +/- 16.076	0.626
	Range	(31–90)	(32–92)	
Type of arch		N (%)	N (%)	0.861
	Type I	51 (51%)	53 (53%)	
	Type II	38 (38%)	35 (35%)	
	Type III	11 (11%)	12 (12%)	
Tortuosity index		1.09 (0.623)	1.10 (0.081)	0.807
	CI^a^	1.08–1.11	1.09–1.12	
Curvature ratio		1.00 (0.120)	1.00 (0.012)	0.411
	CI^a^	1.00–1.00	1.00–1.00	
Maximum Tortuosity		24.58 (6.854)	24.65 (6.343)	0.960
	CI^a^	23.49–26.21	23.39–25.91	
Level of Vertebrae		5	5	1.000
	Range	5–12	5–12	
Minimum Angulation		1.21 (1.20)	1.29 (1.183)	0.449
	CI^a^	0.97–1.45	1.06–1.52	
Level of Vertebrae		11	11	1.000
	Range	7–12	8–12	
Zone of max angulation		4A	4A	0.821
	Range	4A – 4D	4A – 4D	
Group of angulation		Low	Low	0.862
	Range	Low–Moderate–High	Low–Moderate–High	
Percentage of patients with high tortuosity		0 (0%)	0 (0%)	

*P-value is significant when p < 0.05

^a^CI: Confidence Intervals.

For the intra- and interobserver variability we used the ICC. The ICC is normally between 0 and 1; an ICC close to 1 indicates high similarity between values. The ICC was above 0.80 for the tortuosity index, curvature ratio, maximum angulation, the level of vertebrae of the maximum angulation and the zone of the maximum angulation.

## Discussion

The physiological effect of aging on the DTA is inevitable. Knowing that the aorta becomes more tortuous and longer with age can help to improve future generations of stent grafts to obtain less complications and better outcomes.

When Ishimaru introduced the classification of the thoracic aorta in 1996, zone 4 (the DTA) was divided into different zones correlated to the thoracic vertebras[10]. This article shows that zone 4 does not always start with thoracic vertebra 4. As a new classification for the aortic arch was proposed by Marrocco-Trischitta et al. there is no clear classification on zone 4. In the literature tortuosity is an important risk factor for the occurrence of endoleaks in the DTA, including the distal type[13,14,16]. As the human body is becoming smaller, the aorta length can become even longer[1], and therefore with a high tortuosity (> 60 degrees) can occur with age. As tortuosity in the DTA has not been investigated yet, this study contributes to future planning of TEVAR to obtain positive results during follow-up.

In this study we looked at the type of arch in < 65y and ≥ 65y old people. Most people in the < 65y group had a type 1 arch, where people in the ≥ 65y group had a balance between the different types, see Fig 5. The reason for this phenomenon can be multifactorial. One of the factors might be the decrease of elastin and increase of collagen in the aortic wall, which comes with aging and the anatomy of the aorta changes[6]. Secondly, aortic elongation in combination with the fixation of the supra-aortic trunks may account for entry tear formation[19].

The tortuosity index, curvature ratio and the maximum tortuosity are higher in the ≥ 65y group. An explanation as previously mentioned; with aging the human body shrinks, but the aorta is getting longer[1]. A study by Craiem et al. shows that the volume, the diameter and the length of the thoracic aorta increases with age[2]. The curvature radius increased too, but the tortuosity was decreasing, which is in contrast with our findings. This difference might be because of the their different way of measuring tortuosity. Likewise, the maximum degrees of tortuosity is higher in ≥ 65y old people, which can be explained the same way[1]. The level of highest tortuosity is in the ≥ 65y group more distal in the DTA.

Similar to Rylski et al. this study shows that there is no difference in tortuosity and angulation between males and females[20]. A recent study by Tawfik et al. was conducted to define the relation between age and gender on tortuosity[21]. The methodology was similar and the outcome was the same.

We showed that the aorta is more tortuous in older patients, who usually benefit from endovascular repair. However, they do have a higher risk of complications after TEVAR[22,23]. Previously it has been confirmed that a type 1b endoleak is associated with aortic tortuosity[13,14,16]. The findings of this study can declare the higher risk of complications and help with future planning for TEVAR.

Limitations of this study are that only the physiological geometry has been analyzed. The influence of cardiovascular diseases has not been taken into account, whilst this can be of importance. Also the influence of other risk factors for atherosclerosis (i.e. Diabetes Mellitus) were not examined and tested in a regression model. Furthermore, the cross-sectional study design is a limitation. Future studies with a longitudinal design could be informative. Nevertheless, our findings support previous work and so adds to the existing literature in providing some support for the natural evolution of aortic geometry.

Conclusion, the tortuosity and curvature of the DTA increase significantly with age. This acknowledgment is useful for further research on the descending thoracic aorta and to understand the natural development with age, when tortuosity is becoming more important in the planning and follow-up strategy of TEVAR.

## Abbreviations

DTA: descending thoracic aorta
TEVAR: thoracic endovascular aortic repair
CT: computed tomography
LSA: left subclavian artery
ICC: intra correlation coefficient
